# A simple approach to the synthesis of Cu_1.8_S dendrites with thiamine hydrochloride as a sulfur source and structure-directing agent

**DOI:** 10.3762/bjnano.6.90

**Published:** 2015-04-01

**Authors:** Xiaoliang Yan, Sha Li, Yun-xiang Pan, Zhi Yang, Xuguang Liu

**Affiliations:** 1 College of Chemistry and Chemical Engineering, Taiyuan University of Technology, Taiyuan 030024, P. R. China; 2 College of Textile Engineering, Taiyuan University of Technology, Taiyuan 030024, P. R. China; 3 School of Chemistry and Chemical Engineering, Hefei University of Technology, Hefei 230009, P. R. China; 4 College of Physics and Optoelectronics, Taiyuan University of Technology, Taiyuan 030024, P. R. China

**Keywords:** biomaterials, crystal growth, crystal structure, Cu_1.8_S dendrite, hydrothermal

## Abstract

A facile, green and environmental-friendly method for preparing Cu_1.8_S dendrites was developed. Copper nitrate and thiamine hydrochloride were selected as the starting materials in the water phase under hydrothermal conditions. No addition of a surfactant or a complex reagent was required for the synthesis of the Cu_1.8_S dendrite structures. Thiamine hydrochloride was employed as a sulfur source and structure-directing agent. The growth mechanism of Cu_1.8_S is tentatively discussed based on the experimental and computational results.

## Introduction

Recently, Cu_1.8_S with a unique structure has attracted great attention due to its versatile applications in solar cell, electrochemistry, catalysis, and as a gas sensor [1–5]. Many strategies have been developed to prepare Cu_1.8_S. A solvent-mediated methodology was employed to synthesize highly crystalline Cu_1.8_S by element copper and sulfur at room temperature [4]. Polycrystalline Cu_1.8_S powder was prepared by mechanical alloying and subsequent spark plasma sintering and it exhibited excellent thermoelectric properties as a p-type sulfide [5]. An aqueous ammonia assisted approach was developed for the synthesis of Cu_1.8_S with triangular and rod-like shapes from sulfur powder [6]. Lim et al. found that copper sulfide dendritic structures could be obtained at high ethylenediamine and low tributylphosphite concentrations by using a copper(I) thiobenzoate (CuTB) precursor [7]. In general, sulfur, Na_2_S_2_O_3_, mercaptan and thiourea are used as sulfur sources and an additional structure-directing agent is needed for producing metal sulfides with a unique structure [6,8–10]. These sulfur sources and the byproducts are toxic and harmful to the health and the environment. Thus, a simple, inexpensive, and efficient approach for the environmental-friendly preparation of metal sulfide nano/micro-materials is sought for.

Biomolecules have been widely used as a sulfur sources and structure-directing agents in the synthesis of metal sulfides [11–12]. Kim et al. used 2-mercaptoethanol to synthesize high-aspect ratio and single-crystalline nanowires of Bi_2_S_3_ without a template [11]. Li et al. demonstrated that L-cysteine could assist the formation of snowflake-like patterns and flower-like microspheres as well as porous hollow microsphere CuS structures [12]. Thiamine, abundant and inexpensive, contains one sulfur atom and is supposed to be used as a sulfur source. In addition, the functional groups in thiamine may play an important role in the oriented growth of copper sulfide. To the best of our knowledge the application of thiamine hydrochloride, an abundant and cheap biomolecule, and copper nitrate in water for the growth of Cu_1.8_S with a unique structure has not been reported. It was found that thiamine hydrochloride is a good source of sulfur. Moreover, the functional groups in thiamine hydrochloride can help to orient the growth of uniquely structured Cu_1.8_S.

## Results and Discussion

**Figure 1:**
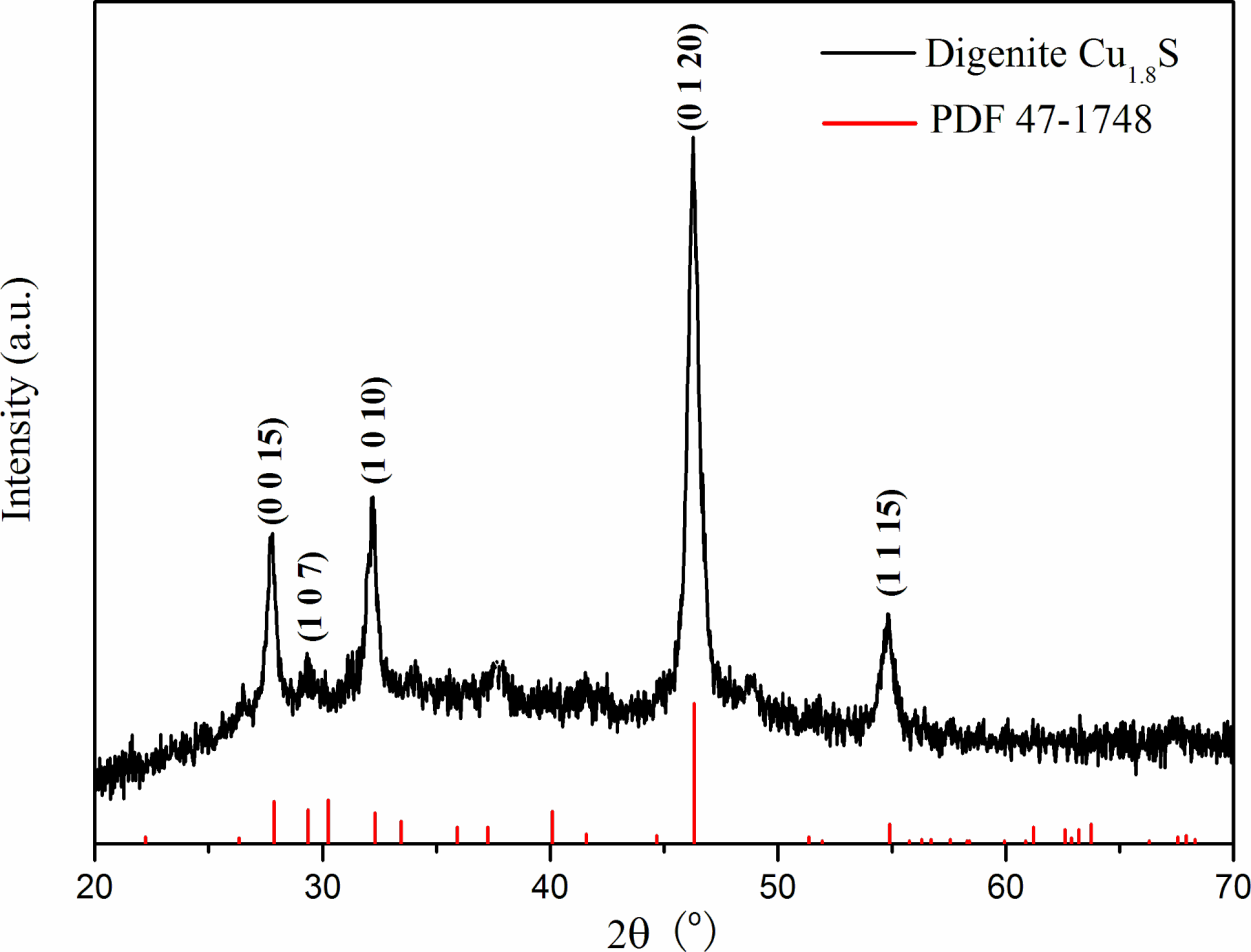
Powder XRD pattern of Cu_1.8_S synthesized after a reaction time of 24 h.

The XRD pattern of the obtained product is shown in Figure 1. Five diffraction peaks are indexed to the digenite Cu_1.8_S phase (JCPDS card, File No. 47-1748). The absence of peaks corresponding to other phases of copper sulfide, such as CuS, Cu_1.75_S, Cu_1.95_S, Cu_2_S, and materials related to the precursors and copper oxides indicates the purity of the product. The product is crystalline, as reflected by the strong and sharp diffraction peaks. These results implied that the digenite Cu_1.8_S phase was obtained from thiamine hydrochloride and the copper precursor under hydrothermal conditions.

**Figure 2:**
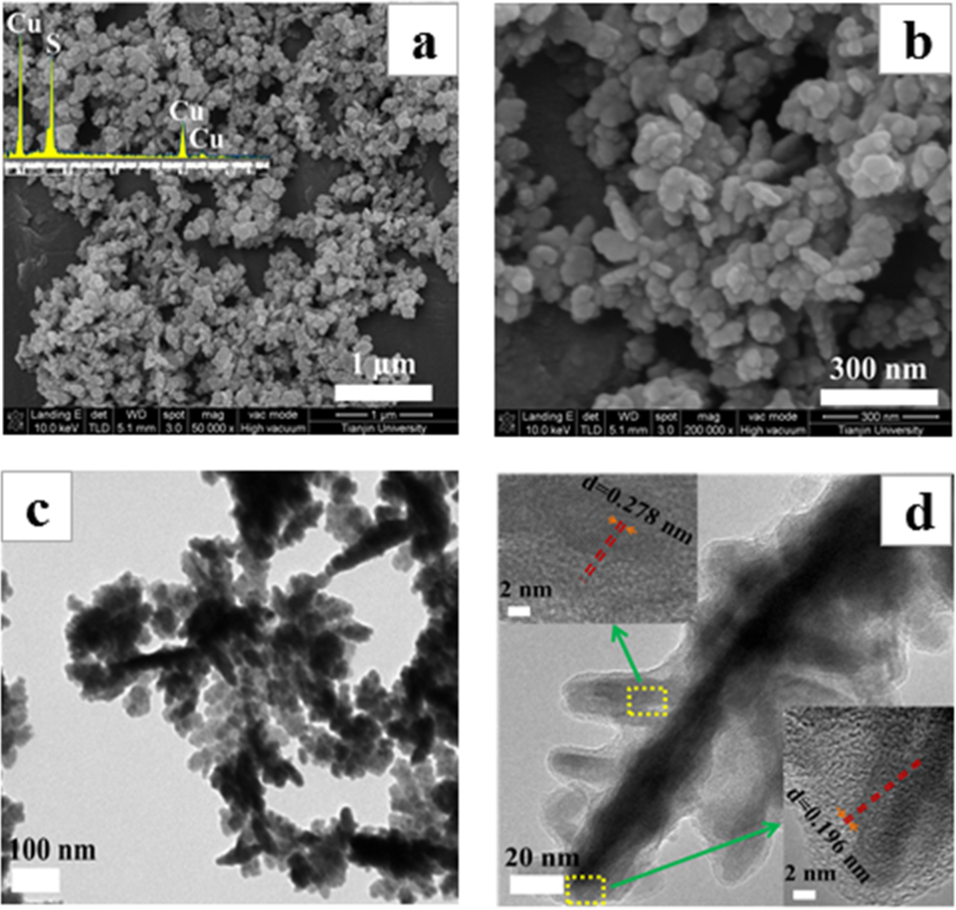
SEM images (a), (b) with EDX analysis, TEM image (c), and high-resolution TEM image (d) of Cu_1.8_S synthesized after a reaction time of 24 h.

The SEM images of the sample synthesized from thiamine hydrochloride and the copper precursor under hydrothermal conditions exhibit short rod-like structures as shown in Figure 2. The EDX analysis confirms that the atomic ratio of Cu:S in the sample is about 1.8:1. This is well-consistent with the result of the XRD analysis, and indicates a pure phase of Cu_1.8_S. Cu_1.8_S with dendritic structures can be clearly seen in the TEM images (Figure 2d). The size and diameter of the trunk of the dendritic structure are 100–300 nm and 30–50 nm, respectively. An inset of Figure 2d displays the high-resolution TEM image of the tip position of dendrites (main trunk and secondary trunk), and the observed lattice spacing of 0.196 and 0.278 nm match with the (0 1 20) and (1 0 10) planes of Cu_1.8_S, respectively. It can be concluded from the analysis that the main trunk of a Cu_1.8_S dendrite grows along the (0 1 20) direction.

To understand the formation mechanism of the Cu_1.8_S dendrite, we investigated the morphology evolution of Cu_1.8_S as a function of the hydrothermal process time. Burford et al. reported that the functional groups in biomolecules, e.g., –NH_2_, –COOH, and –S–, are strongly inclined to interact with inorganic cations based on a mass spectrometry study [13]. This indicates that metal ions could interact with biomolecules to form stable complexes. In this experiment, copper nitrate and thiamine hydrochloride is dissolved in water to form a mixture in which Cu^2+^ ions coordinate with thiamine hydrochloride to form a complex. When the mixture was sealed and kept at 180 °C under high pressure, the complexes decompose and Cu_1.8_S nuclei are produced, as described by Equation 1:

[1]
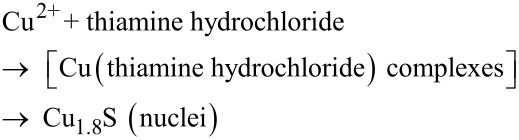


**Figure 3:**
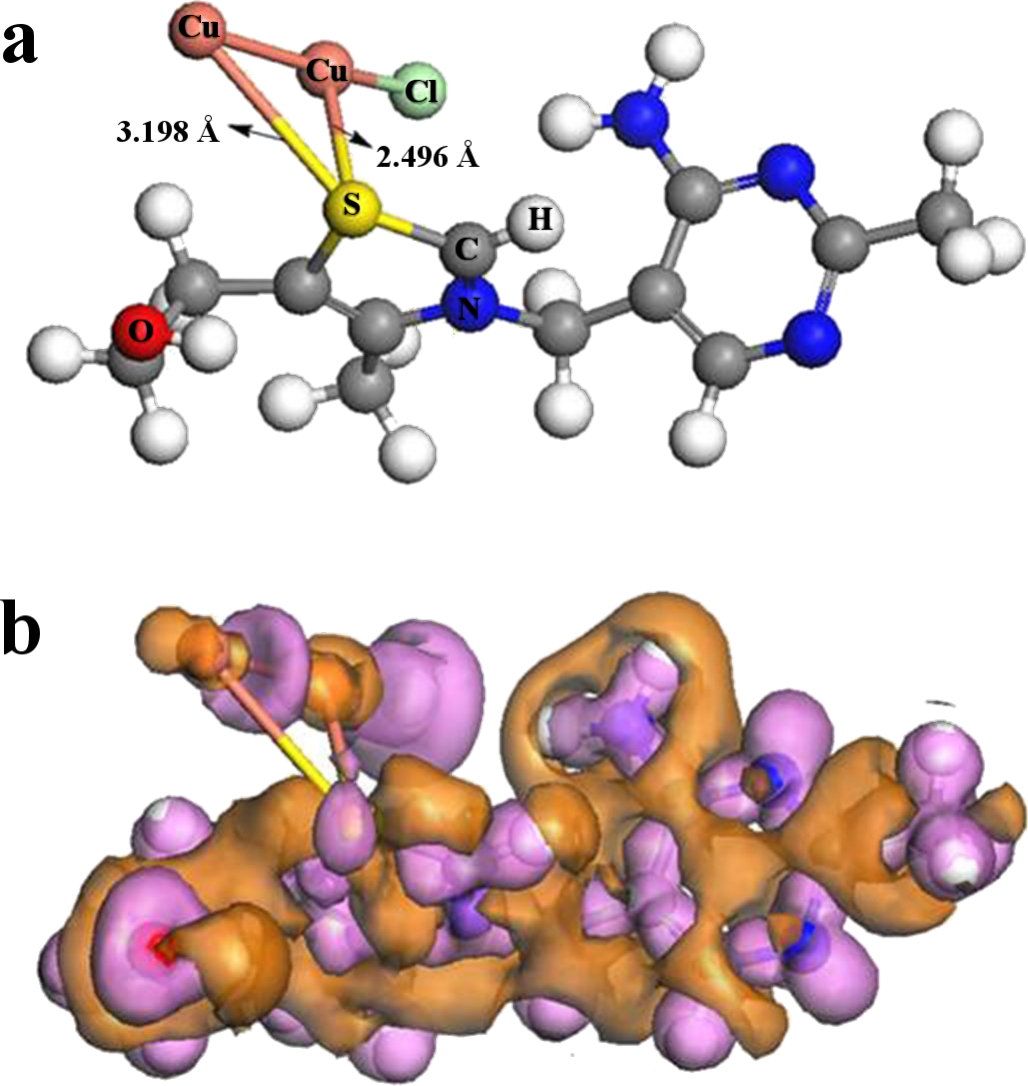
The optimized structure (a) and deformation density (b) of the cluster.

To give a detailed description of the complex, we performed density functional theory (DFT) calculations with a cluster model. In this cluster model, two Cu atoms were added to C_12_H_17_ClN_4_OS·HCl to represent possible interactions. The geometry optimization of the cluster was carried out by using the DMol^3^ package [14]. The Perdew–Burke–Ernzerhof (PBE) functional and double numerical basis set with polarization functions (DNP) were employed [15]. As can be seen in Figure 3a, the two Cu atoms could form two chemical bonds with S, exhibiting a distorted local tetrahedron configuration. The bond lengths of Cu–S are 2.496 and 3.198 Å, respectively, which indicates that the interaction between Cu and S is significant. In particular, the Mayer bond orders of Cu–S bonds are 0.402 and 0.138, which means that the Cu–S bonds exhibit a covalent component. In fact, such an interaction between Cu and S can also be understood from the deformation density, as shown in Figure 3b. The DFT results show that an interaction between Cu and S indeed exists.

**Figure 4:**
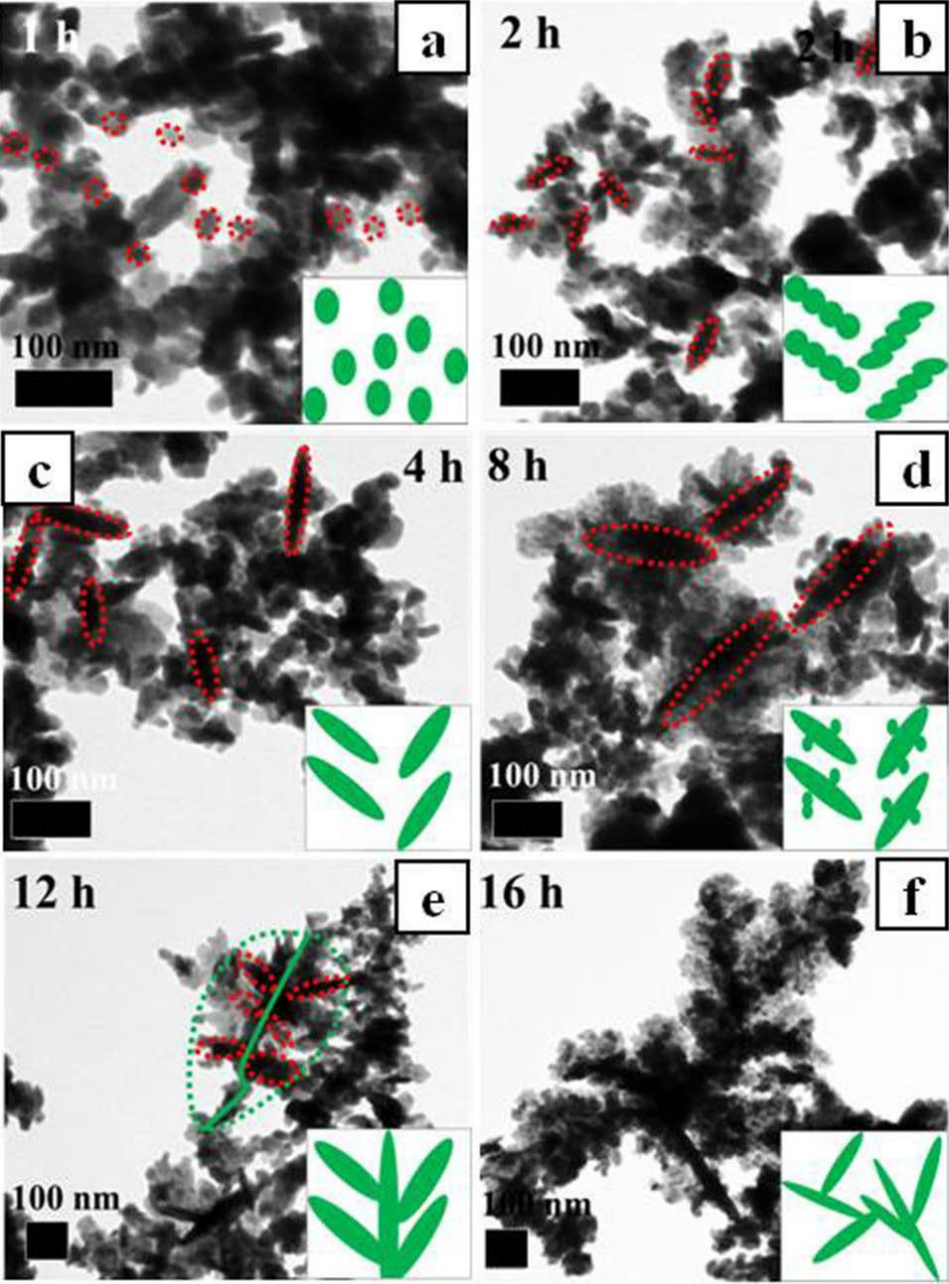
TEM images and schematic illustrations (bottom right corner) of Cu_1.8_S dendritic structure after different treatment times: 1 h (a), 2 h (b), 4 h (c), 8 h (d), 12 h (e), 16 h (f).

Figure 4 shows the morphological changes of the Cu_1.8_S dendritic structure in dependence on different treatment times. The Cu_1.8_S nuclei grew into nanoparticles after a reaction time of 1 h under hydrothermal conditions, as shown in Figure 4a. With the reaction time increasing to 2 h and further to 4 h, the nanoparticles self-assembled into rod-like structure (Figure 4b,c). A large number of petiole-like structures were formed and surrounded by small nanoparticles after 8 h of reaction time (Figure 4d). When the reaction time prolonged to 12 h, leaflet morphology was observed (Figure 4e). Longer reaction time (16 h) resulted in Cu_1.8_S with a dendritic structure, as shown in Figure 3f. After a reaction time of 24 h under hydrothermal conditions, the perfect dendrite was obtained through Ostwald ripening. The secondary and third level dendrite appears and leads to the formation of a dendritic net structure. Most of the product evolved into fully 2D dendritic structure, as shown in Figure 2c.

Li et al. and Liu et al. have discussed the growth process and revealed the mechanism of metal sulfide synthesis by using L-cysteine and L-methionine, respectively [12,17]. They suggested that the growth process of metal sulfide crystals exhibit two stages: an initial nucleating stage and a subsequent growth stage. Metal cations reacted with biomolecules to form a complex, then the coordinate bonds ruptured because of the high reaction temperature. In the present system, thiamine hydrochloride plays a significant role in the synthesis of Cu_1.8_S dendrite. Firstly, it is an environmental-friendly and cheap sulfur source. Secondly, the functional group (–C–S–C–) in the Cu (thiamine hydrochloride) complexes breaks at 180 °C and releases free S^2−^ ions in water. The Cu^2+^ ions interact with free S^2−^ ions and produce Cu_1.8_S nuclei. Then, due to the larger amount of thiamine hydrochloride in comparison with that of copper nitrate, the excessive thiamine hydrochloride in the system probably acts as a structure-directing agent for the self-assembly of the nuclei into dendritic structures. This is consistent with the result that the presence of L-cysteine was in favor of the formation of Cu_3_BiS_3_ dendrites [16].

## Conclusion

A hydrothermal process was used for a facile and environmental-friendly synthesis of Cu_1.8_S with thiamine hydrochloride as a sulfur source and water as the solvent. Cu_1.8_S dendrites were obtained after a reaction time of 24 h. The length of the dendritic structure ranges from 100 to 300 nm and its diameter from 30 to 50 nm. The formation process of the Cu_1.8_S dendrite was explored by TEM observations at different reaction times. The DFT results revealed that interactions between Cu and S indeed exists. It was found that the formation of the Cu_1.8_S dendrites probably proceeded by the following process: i) Cu (thiamine hydrochloride) complexes were first obtained; ii) Cu_1.8_S nuclei were produced from the decomposition of the complexes; iii) as-synthesized nanoparticles self-assembled into dendrite. The investigated method with thiamine hydrochloride as a sulfur source for the preparation of Cu_1.8_S dendrite in the present work can probably be employed for the production of other metal sulfides.

## Experimental

The chemicals, including copper nitrate and thiamine hydrochloride (Tianjin Kemiou Chemical Agent Factory) were of analytical grade and used without further purification. In a typical procedure, 0.5 g thiamine hydrochloride was dissolved in 60 ml distilled water, then 0.073 g Cu(NO_3_)·3H_2_O was added to the solution to give the final molar ratios of [thiamin]:[Cu(NO_3_)·3H_2_O] = 5:1, and the solution was stirred for 10 min. Finally, the resulting mixture was sealed into a 100 mL Teflon-lined stainless-steal autoclave, and kept at 180 °C for 24 h. After the solution cooled down to room temperature, the obtained material was washed with water and alcohol for several times, and dried at 60 °C in vacuum.

X-ray diffraction (XRD) analysis was performed with a Rigaku D/MAX-2500 V/PV spectrometer by using Cu-Kα radiation (40 kV and 200 mA) at a scanning speed of 4°/min over the 2θ range of 20–70°. Scanning electron microscopy (SEM) images were recorded with the Hitachi Field Emission Scanning electron microscope S4800. TEM analyses were performed on a Philips Tecnai G^2^ F20 system operated at 200 kV.
